# Cross-cultural diversity analysis: traditional knowledge and uses of freshwater fish species by indigenous peoples of southern Punjab, Pakistan

**DOI:** 10.1186/s13002-022-00573-1

**Published:** 2023-01-09

**Authors:** Khalid Javed Iqbal, Muhammad Umair, Muhammad Altaf, Tanveer Hussain, Rana Manzoor Ahmad, Sayed Muhammad Zain Ul Abdeen, Andrea Pieroni, Arshad Mahmood Abbasi, Shahzad Ali, Sana Ashraf, Naila Amjad, Abdul Majid Khan, Rainer W. Bussmann

**Affiliations:** 1 Department of Zoology, The Islamia University of Bahawalpur, Bahawalpur, Pakistan; 2 College of Life Science, Zhejiang Normal University, Jinhua, 321004 China; 3 Department of Forestry, Range and Wildlife Management, The Islamia University of Bahawalpur, Bahawalpur, Pakistan; 4 Department of Zoology, GC University Lahore, Lahore, Pakistan; 5 University of Gastronomic Sciences, Piazza Vittorio Emanuele II 9, 12042 Pollenzo, Italy; 6 Department of Medical Analysis, Tishk International University, Erbil, 4401 Iraq; 7 Department of Environment Sciences, COMSATS University Islamabad, Abbottabad Campus, Abbottabad, 22060 Pakistan; 8 Department of Zoology, University of Lahore, Sargodha Campus, Sargodha, Pakistan; 9 Department of Zoology, University of the Punjab, Lahore, Pakistan; 10 Department of Ethnobotany, Institute of Botany and Bakuriani Alpine Botanical Garden, Ilia State University, 0105 Tbilisi, Georgia; 11 Staatliches Museum Für Naturkunde, Erbprinzenstrasse 14, 76133 Karlsruhe, Germany

**Keywords:** Pakistan, Medicinal, Raho, Traditional knowledge, Ethnozoology

## Abstract

**Background:**

Fisheries have tremendous cultural and educational importance in human societies. The world is undergoing fast environmental and cultural changes, and local knowledge is being lost. Understanding how people interpret environmental change and develop practices in response to such change is essential to comprehend human resource use. This study was planned with the intent to document and conserve the knowledge about the uses of the freshwater fish fauna among the residents in South Punjab, Pakistan.

**Methods:**

Semi-structured interviews and questionnaires were conducted to collect data from informers (*N* = 88). Principal component analysis, relative frequency citation, fidelity level, relative popularity level, rank-order priority, and similarity index were used to analyze the fish data.

**Results:**

Overall, a total of 43 species of fishes were utilized in the study region, but only 26 species were utilized ethnomedicinally to treat a variety of illnesses such as asthma, body weakness, burn, chicken pox, cold, cough, eyesight, hepatitis, impotence, joint pain, night blindness, skin burn, spleen treatment, stomach infection, and weakness. The uses of fishes were analyzed employing various indices. The highest use value (UV) of 0.86 was calculated for spotted snakehead (*Channa punctata*), whereas the lowest UV of 0.05 was attained by karail fish (*Securicula gora*). Moreover, *Channa punctata*, *Cyprinus carpio*, *Labeo rohita*, *Oreochromis niloticus*, *Wallago attu*, *Hypophthalmichthys molitrix*, *Rita rita*, *Sperata seenghala*, *Notopterus notopterus*, *Labeo dyocheilus*, *Systomus sarana*, *Puntius punjabensis*, *Securicula gora*, *Ompok bimaculatus*, and *Ompok pabda* were the most popular species with RPL = 1.0. Out of the total, 20 species had a “zero” similarity index, while the ethnomedicinal use of 12 species (i.e., *Labeo dyocheilus*, *Labeo boggut*, *Systomus sarana*, *Puntius punjabensis*, *Aspidoparia morar*, *Securicula gora*, *Crossocheilus diplochilus*, *Mastacembelus armatus*, *Ompok bimaculatus*, *Ompok pabda*, *Labeo gonius*, and *Sperata seenghala*) was documented for the first time for a variety of diseases (i.e., body weakness, stomach infection, skin burn, joint pain, impotence, asthma, spleen treatment, and chicken pox).

**Conclusion:**

Our findings showed that the local people of the study area hold noteworthy traditional knowledge about the medicinal and cultural uses of fish species. Furthermore, a comprehensive analysis of active chemicals and in vivo and/or in vitro activities of chemicals derived from ichthyofauna with the highest FC as well as UVs could be interesting for research on new drugs.

**Supplementary Information:**

The online version contains supplementary material available at 10.1186/s13002-022-00573-1.

## Introduction

Aquatic resources have long been important for humans for a wide variety of uses. Fish is an important source of protein and vitamins, and fish management has always been crucial. Freshwater fish have also had great cultural, especially as part of cultural foods commemorating specific calendar occasions. It is critical to understand the history of these connections in order to research the interactions between fish and humans [1]. Environmental change and over-exploitation have already led to a decline in fish supplies at the Kenyan coastline [2, 3].

Rivers, lakes, and streams play a significant role in the Asian landscape, providing a diverse range of flora and fauna. There are numerous fish species in Asian waters, and they have also been of great value to the countryside market, producing foodstuffs both natively and for the rising metropolitan populace [4–7]. Fishermen's perceptions of fish behavior and ecology may give useful information to aid the conservation and sustainable management of these rivers, lakes, and streams, including the possible consequences of climate change. Ethnoichthyology includes aspects of conservation and cultural behavior [8], as well as ethnotaxonomy [9], with local names of species generally based on sound, environment, habitat, myth, morphological characteristics, and social links [10]. Vernacular names for fish are important sources for ethnobiologists, anthropologists, linguists, and government officials and should be documented [11].

Scientists have documented more than 32,000 species of fishes from the world [12], among them more than 746 species were recorded from Pakistan in 2022 [13]. Ethnobiologists documented dynamic interrelationships between human and the surrounding biota [14, 15] and also noted human impact on the ichthyofauna [16]. Fishes have lots of cultural uses as tools [17], food [10, 18, 19], medicine [18–20], and for trade [17]. The traditional use of freshwater fishes has, however, never been documented before in southern Punjab. Accordingly, this study was designed to record and conserve the traditional information and knowledge about freshwater fish species and their cultural and medicinal uses by the people residing in South Punjab, Pakistan. We endeavored to answer the following questions: (1) How many freshwater fish species are employed as therapeutic medication in the healthcare system of southern Punjab, Pakistan? (2) Which species are the most frequently used in southern Punjab? (3) What are the key points to consider when using fish fauna for medicinal and cultural purposes? (4) What are the basic socioeconomic factors influencing the use of fish species for medicinal and cultural purposes (gender, educational status, occupation, and ethnicity)? (5) How can we conserve the traditional information and knowledge about using fish species for medicinal and cultural purposes?

## Materials and methods

### Study area

South Punjab is a region in the Punjab Province with a diverse geography and climate [21]. The residents in this region are mostly illiterate and extremely poor and rely entirely on handcrafted wooden items, agriculture, livestock, and embroideries. The area extends from 71°27′ to 73°15′ east to 29°12′ to 31°15′ north. It has a total size of 105,504 km^2^ [22] and is rich in natural resources due to its diverse geographical nature [23]. The Dera Ghazi Khan, Sahaiwal, Multan, Dera Ghazi Khan, and Ranjanpur tribal areas are in the north, Vahari and Bahawalnagar in the southwest, Bahawalpur in the west, and Sadiqabad and Rahim Yar Khan in the northwest. The climate is arid to semiarid. In the summer, it is quite hot, and the temperature reaches about 45 °C, while in winter, the temperature drops to 2 °C. The area is considered to be one of the hottest regions in the country. The hottest months are May, June, and July. The average rainfall ranges between 100 and 180 mm and approximately half of the total rainfall falls during the months of July and August [22]. Irrigated areas support a wide range of food and fodder crops [24].

The ethnic makeup of the area is quite diverse, with Saraiki, Punjabi, and Baloch people predominating. The Saraiki are the strongest ethnic group in South Punjab and are extensively spread over the area [25]. The population of South Punjab are Muslim. The predominant native language spoken in South Punjab is Saraiki, which is generally spoken in most sections of the province [26], Punjabi is also commonly spoken in the eastern portion of the Bahawalpur Division, and Urdu is frequently used as an official language [27]. The location is extremely remote from urban areas and has rough barren land. Residents' socioeconomic status is poor, and they lack access to health care. The roads and other infrastructure are in disrepair, and many locals rely on farming, livestock, and local businesses to survive. Only a few are educated and work for the government, and only a few serves in other counties.

### Fish identification and documentation

**Fig. 1:**
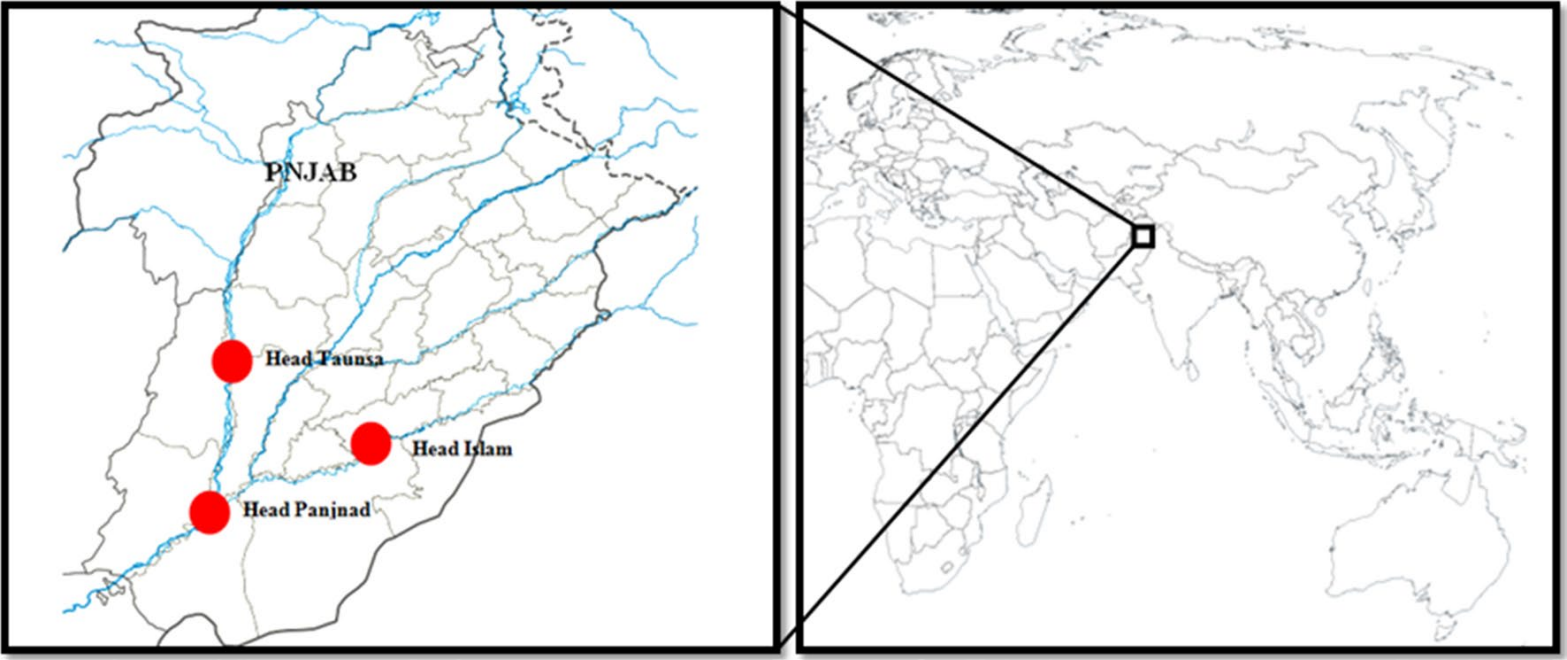
Map of Punjab along with study areas, i.e., head Taunsa, head Islam, and head Panjnad

Fish data were collected from February 2021 to January 2022 in selected subareas of head Taunsa, head Islam, and head Panjnad (Fig. 1), using semi-structured interviews and group discussions with 88 collaborators including questions on the profile of participants, vernacular names of fishes, cultural uses (i.e., rituals, tool, entertainment, and food) as well as ethnomedicinal use of fishes, after obtaining oral prior informed consent. Additional file 1: Table S1 presents the scientific, common and local names, and conservation status of these species. Participants’ age, gender, educational status, occupation, and ethnicity were collected as demographic data. The questionnaires were first written in English and then translated into Saraiki, Punjabi, and Urdu. Before the start of survey work, proper permission was obtained from the IRB, Department of Zoology, The Islamia University of Bahawalpur, Pakistan.

Interviews were conducted during the daytime, and specimens (e.g., pictures, carcasses, etc.) were collected during different visits. Collaborators were gathered randomly [28, 29]. Some images of fish species were also included in the questionnaires. Collaborators had a minimum age of 18 years and a maximum age of 70 years.

Fish species in the study area were identified directly by the locals and confirmed through photographs requested in the questionnaire and sent by e-mail or Facebook messaging. The book of “Freshwater Fishes of Pakistan” was studied for the classification of species of fishes of southern Punjab [30]. The scientific species and genus were checked by the fish database catalog (https://tropicalfreshwaterfish.com/data/Pakistan.htm) in the current study's scientific classification systems [13].

### Quantitative analysis

The cultural and ethnopharmacological data of fish species were documented and analyzed with diverse indices, i.e., relative frequency citation, fidelity level, relative popularity level, rank-order priority, and similarity index.

#### Relative frequency of Citation (RFC)

The RFC shows the worth of all fishes of southern Punjab [31, 32] and was calculated as follows [33]:

$${\text{RFC}} = {\text{FC}}/{\text{N}}\left( {0 < {\text{RFM}} > 1} \right)$$

where “FC” is the citation of the collaborator and N is the number of all collaborators.

#### Use value (UV)

UV was calculated as:

$$UV = \sum U/n$$

where *UV* is the use value of a fish species, *n* is the numeral of citations per fish species, and *U* is the number of collaborators [34, 35].

#### Relative popularity level (RPL)

This index is calculated as the proportion of cultural use reports divided by the mean value of reports. All fish were classified into two groups, i.e., popular and unpopular. The RPL assumes a value ranging from “zero” to “one,” with “zero” indicating no cultural uses by a specific species of fish and “one” indicating the entire popularity of a specific species of fish. The “RPL” value for fish species in the “unpopular group” is less than one [36, 37].

#### Fidelity level (FL)

FL was calculated as follows [37, 38]:

$${\text{FL }}(\% ) = I_{{\text{p}}} /{\text{Iu}} \times 100$$

*I*_p_ = number of collaborators who independently cited the importance of a species for treating a particular disease, Iu = total number of collaborators who reported the organism for any given disease.

#### Rank-order priority (ROP)

ROP was calculated as follows [36, 37]:

$${\text{ROP}} = {\text{FL}} \times {\text{RPL}}$$

#### Similarity index (SI)

SI is calculated as [39]:

$${\text{SI}} = S_{{\text{a}}} /T_{{\text{a}}} \left( {0 < {\text{RFM}} > 1} \right)$$

where *S*_a_ = similar noted illness in the previous and present study, *T*_a_ = total noted illness in the present study.

### Statistical analysis

The usage of fish species to treat various diseases was illustrated using “chord diagrams” and the “circlize package” in “R statistical software 3.6.1” [40]. Principal component analysis (PCA) was used employing Past (Version 3.20) statistical software [41].

## Results

### Demography of respondents

**Fig. 2:**
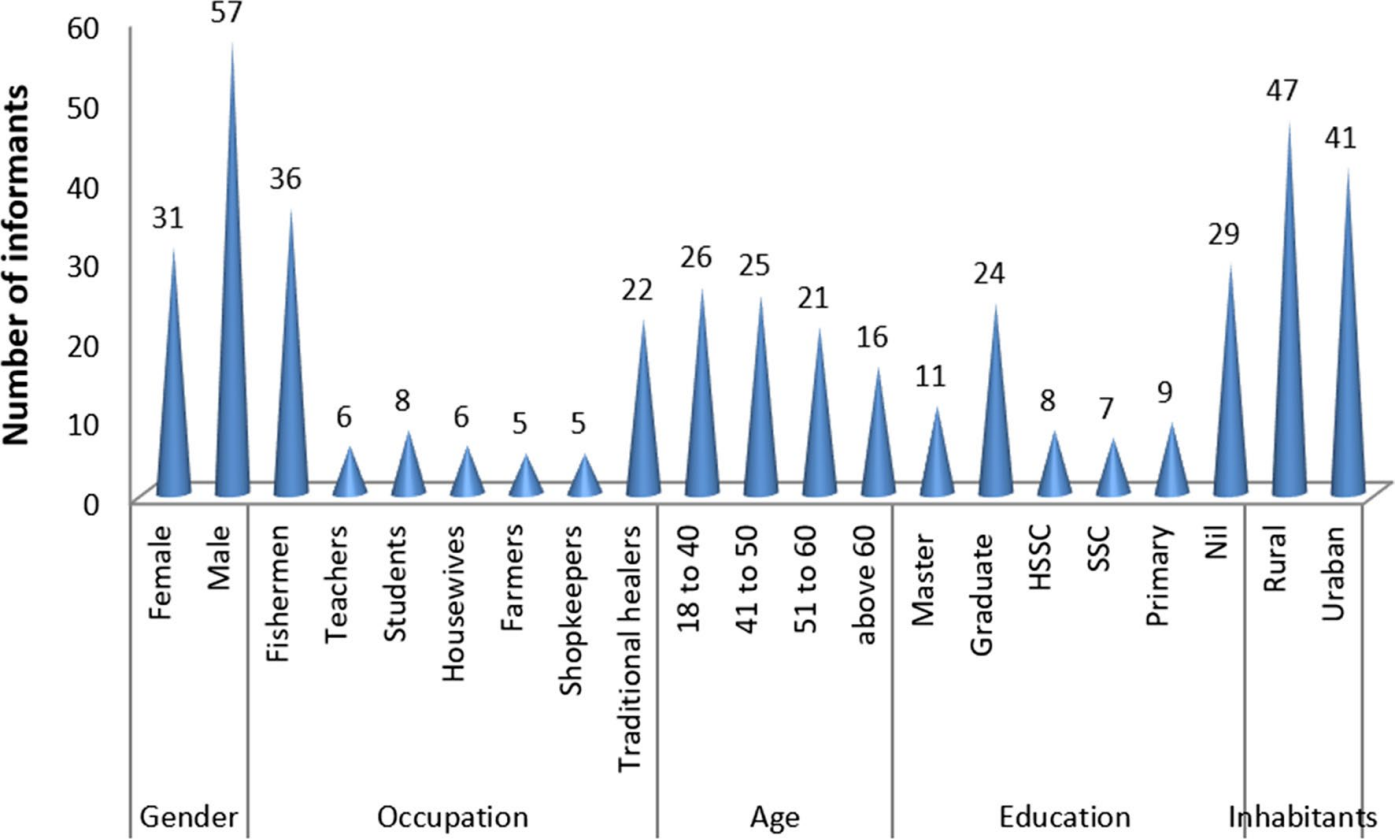
Profile of collaborators

Information on fish use was gathered from men (*n* = 57) as well as women (*n* = 31). Total collaborators (*n* = 88) with ages from 18 to 70 years (Fig. 2). Most of the collaborators (*n* = 59) were educated, having primary (*n* = 9), SSC (*n* = 7), HSSC (*n* = 8), graduate (*n* = 24), and master (*n* = 11) education (11, 5, 12, 4, and 7, respectively). Most of the collaborators (*n* = 47) live in rural areas (Fig. 2).

### Principal component analysis (PCA)

**Fig. 3:**
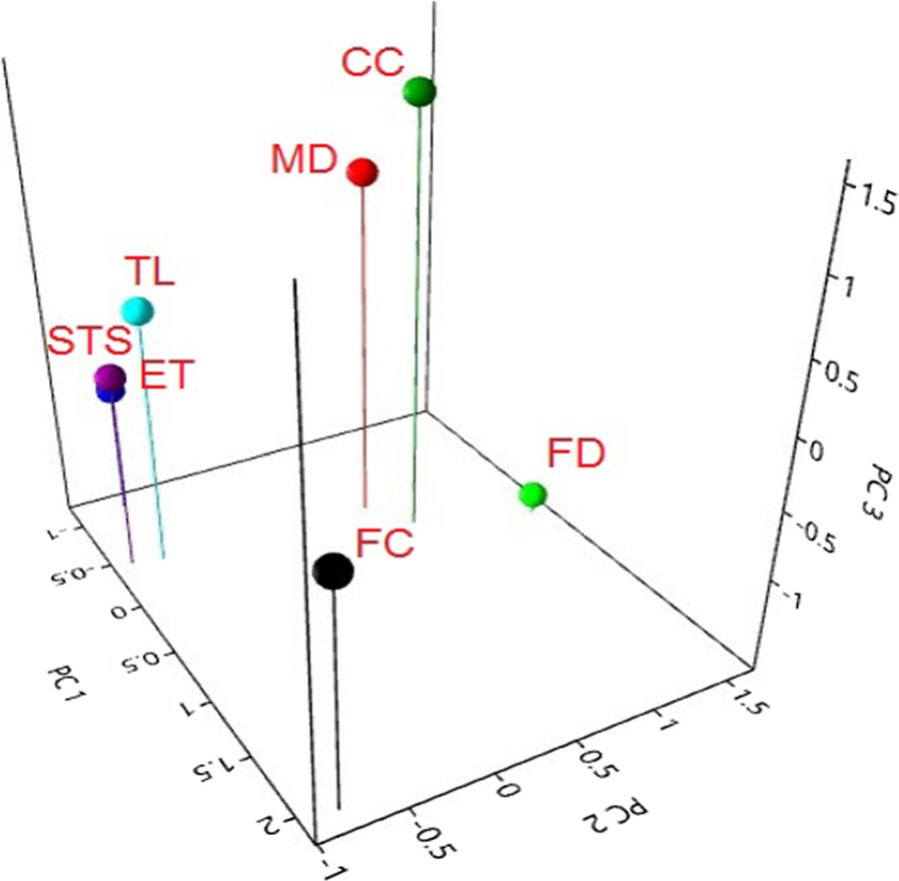
Cultural uses of fishes of southern Punjab analysis through principal component analysis. FC (frequency of citation), MD (medicinal), STS (superstitious/ritual), CC (commercial), TL (tool), ET (entertainment), and FD (food)

**Fig. 4:**
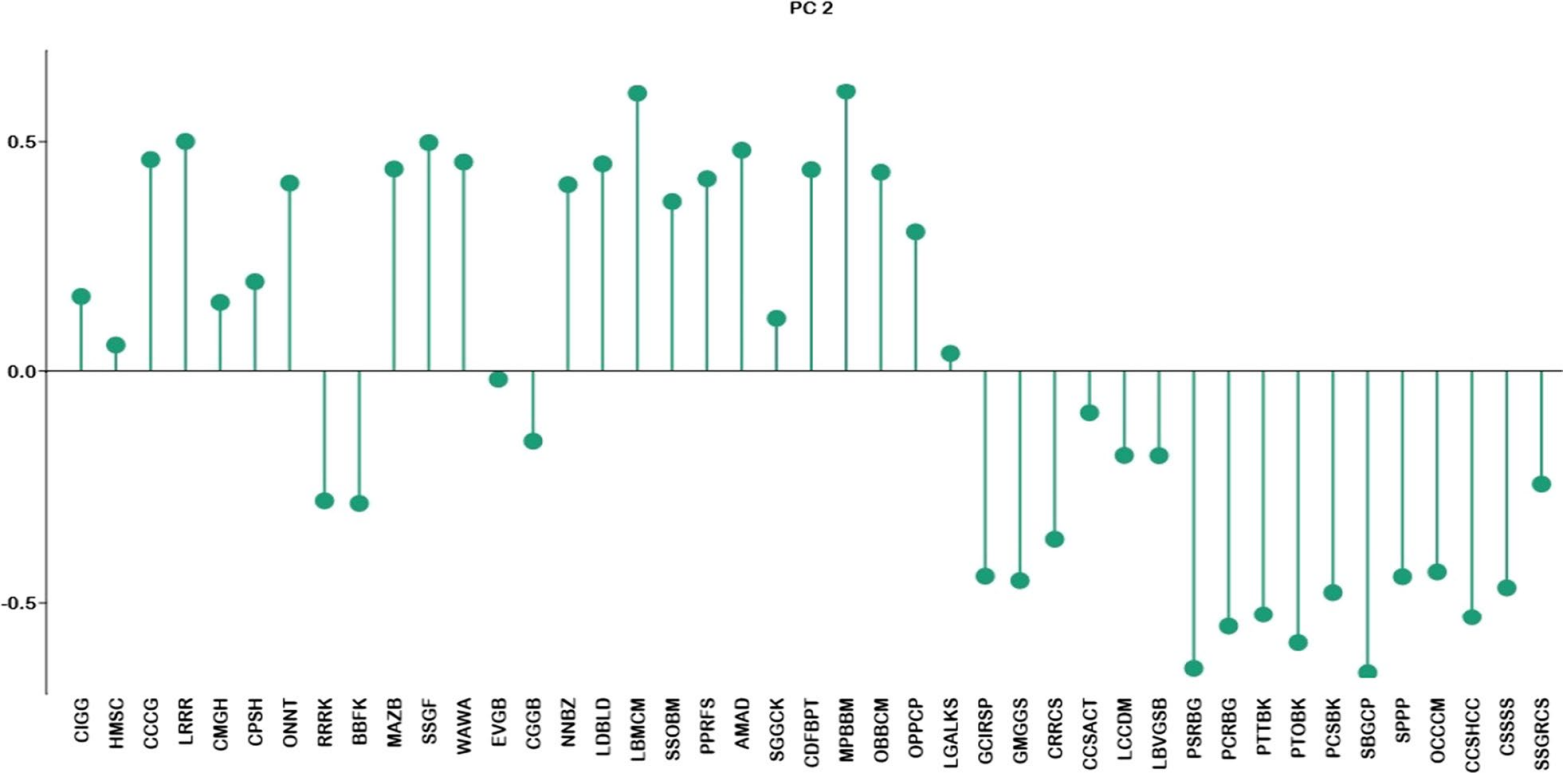
Loadings of variables in PC2, codes are present in Additional file 1: Table S1

The cultural information was examined through PCA (Fig. 3), with support of plot direction in all six variables i.e., FC (frequency of citation), MD (medicinal), STS (superstitious/ritual), CC (commercial), TL (tool), ET (entertainment), and FD (food). The result of the PCA demonstrated the total of whole eigenvalues of the entire fauna. The first eigenvalue was highest (31.8) indicating the highest gradient power in distribution of local information along the component 1 (C1). The first 2 components of the PCA yielded 91% variations in samples (C1: 74.2%; component 2 abbreviated as (C2): 16.8%). These variables were FC (*r* = 2.0552), MD (*r* = −0.31819), STS (*r* = −1.1064), CC (*r* = −0.01245), TL (*r* = −0.41477), ET (*r* = −0.46067), and FD (*r* = 0.25724) which were positively related to PC1, while FC (*r* = −0.79969), MD (*r* = 0.6607), STS (*r* = −0.68906), CC (*r* = 0.844), TL (*r* = −0.70042), ET (*r* = −0.88496), and FD (*r* = 1.5694) were negatively related to PC2 and (*r* = 0.39521) was positively related with C2 (Fig. 4).

### Frequency of citation (FC)

The species of fishes reported by the maximum number of collaborators were commonly utilized to cure various illnesses. *Labeo dyocheilus* had a maximum FC (79), followed by *Notopterus notopterus* and *Clupisoma garua* (79 and 51, respectively) (Table 1).

**Table 1:** Statistical analysis of ethnopharmacological uses of fishes of South Punjab

Sr. No	Name	Code	Diseases	BPU	MOA	Ip	Iu	UV	FL	RPL	ROP	Previously reported diseases	References	SI
1.	*Ctenopharyngodon idella* (Valenciennes, 1844), Grass carp, Grass carp	CIGG	**Eyesight**, cough, cold, joint and backbone pain	Whole body	Oral	65	43.00	0.66	66.15	0.98	65.1	Sexual power, joint pain, backbone pain, enhance memory, energy, cold	[10, 75, 76]	0.25
2.	*Hypophthalmichthys molitrix* (Valenciennes, 1844), Silver carp, Silver carp	HMSC	Eyesight, cough, cold, night blindness, **joint and backbone pain**	Whole body	Oral	70	37.00	0.53	52.86	1.00	52.86	Night blindness, fever, eyesight, cough, cold, backbone pain	[10, 17]	0.83
3.	*Cyprinus carpio* (Linnaeus, 1758), Common carp, Gulfam	CCCG	Eyesight, cough, cold, **night blindness**, joint, backbone pain	Whole body	Oral	72	45.00	0.63	62.50	1.00	62.50	Sexual power, overweight, lumbago, erysipelas, memory, energy, cold, CNS	[49, 76]	0.08
4.	*Labeo rohita* (Hamilton, 1822), Rohu, Roho	LRRR	**Eyesight**, cough, cold, joint and backbone pain	Whole body	Oral	61	33.00	0.54	54.10	0.92	50.00	Weakness, rheumatic pain, cold, urine problem, stomachache, enhance memory, energy, sexual power,	[17, 76–79]	0.07
5.	*Channa marulius* (Hamilton, 1822), Great snakehead, Sol	CMGH	**Cough**, impotency	Whole body	Oral	58	7.00	0.12	12.07	0.88	10.61	Hemoglobin, memory, energy, sex power, cure cold, joint pain, rheumatic pain	[17, 20, 77, 78, 80]	0
6.	*Channa punctata* (Bloch, 1793), Spotted snakehead, Dola	CPSH	Impotency, **weakness**	Whole body	Oral	51	44.00	0.86	86.27	0.77	66.67	Malaria, joint pain, cold, sexual power, enhance energy, appetite, body pain, blood purification,	[17, 76, 78, 81, 82]	0
7.	*Oreochromis niloticus* (Linnaeus, 1758), Nile tilapia, Tilapia/Chira machhli	ONNT	**Body weakness**, chicken pox, skin burn	Meat, skin, oil	Oral, topical	73	30.00	0.41	41.10	1.00	41.10	Vision, scorpion bite, abscesses, carbuncle, energy, sexual power, enhance memory	[17, 19, 49, 76]	0
8.	*Rita rita* (Hamilton, 1822), Rita, Khaga	RRRK	Skin burn	Whole body	Oral	69	22.00	0.32	31.88	1.00	31.88	Impotency, cold, joint issues, CNS, joint pain, enhance energy	[17, 76, 77]	0
9.	*Bagarius bagarius* (Hamilton, 1822), Goonch, Foji Khaga	BBFK	Impotency, **joint pain**	Meat	Oral	53	12.00	0.23	22.64	0.80	18.18	Body pain, burns, stomach issues	[78, 83]	0
10.	*Mastacembelus armatus* (Lacepède, 1800), Zig-zag eel, Baam machhali	MAZB	**Impotency**, joint pain	Meat	Oral	74	27.00	0.36	36.49	1.00	36.49	Sexual problems, weakness	[17]	0
11.	*Sperata seenghala* (Sykes, 1839), Giant river catfish, Sangari	SSGF	Chicken pox, **joint pain**	Meat	Oral	69	27.00	0.39	39.13	1.00	39.13			0
12.	*Wallago attu* (Bloch & Schneider, 1801), wallago catfish, Mali	WAWA	Joint pain	Meat, skin, oil	Oral, topical	77	29.00	0.38	37.66	1.00	37.66	Liver tonic, pile, dysentery, memory, sexual issues, joint issues, liver, cold,	[76, 84–86]	0.12
13.	*Eutropiichthys vacha* (Hamilton, 1822), Batchwa vacha, Jhali	EVGB	Hepatitis	Meat	Oral	52	13.00	0.25	25.00	0.79	19.70	Joint pain	[10]	0
14.	*Clupisoma garua* (Hamilton, 1822), Garua bachcha, Bachhwa	CGGB	Joint pain	Meat, skin, oil	Oral, topical	55	11.00	0.20	20.00	0.83	16.67	Joint pain	[10]	1
15.	*Notopterus notopterus* (Pallas, 1769), Bronze featherback, But Pari	NNBFBP	Joint pain	Meat, skin, oil	Oral, topical	71	47.00	0.66	66.20	1.00	66.20	Pain, chicken pox	[87, 88]	0
16.	*Labeo dyocheilus* (McClelland, 1839), Brahmaputra Labeo, Dambra	LDBLD	**Body weakness**, chicken pox	Meat	Oral	79	43	0.54	54.43	1.00	54.43			0
17.	*Labeo boggut* (Sykes, 1839), Minor carp, Mori	LBMCM	Joint pain	Meat, skin, oil	Oral, topical	62	43	0.69	69.35	0.94	65.15			0
18.	*Systomus sarana* (Hamiliton, 1822), Olive barb, Mori	SSOBM	Joint pain	Meat, skin, oil	Oral, topical	68	41	0.60	60.29	1.00	60.29			0
19.	*Puntius punjabensis* (F. Day, 1871), Ray-finned fish, Silver fish	PPRFS	Joint pain	Meat, skin, oil	Oral, topical	74	26	0.35	35.14	1.00	35.14			0
20.	*Aspidoparia morar* (Hamiliton, 1822), Aspidoparia, Dahi Machli	AMAD	Stomach infection	Meat	Oral,	55	19	0.35	34.55	0.83	28.79			0
21.	*Securicula gora* (Hamiliton, 1822), Gora-chela, Karail fish	SGGCK	Skin burn	Meat, skin, oil	Oral, topical	77	4	0.05	5.19	1.00	5.19			0
22.	*Crossocheilus diplochilus* (Heckel, 1838), Fringe barb, Pahari torki	CDFBPT	Joint pain	Meat, skin, oil	Oral, topical	63	38	0.60	60.32	0.95	57.58			0
23.	*Mastacembelus armatus* (Lacepède, 1800), Zig-zag eel, Baam machhali	MPBBM	Joint pain, **impotency**	Meat, skin, oil	Oral, topical	60	42	0.70	70.00	0.91	63.64			0
24.	*Ompok bimaculatus* (Bloch, 1793), Butter Catfish, Mali	OBBCM	**Asthma**, spleen treatment	Meat	Oral	72	24	0.33	33.33	1.00	33.33			0
25.	*Ompok pabda* (Hamiliton, 1822), Pabdah Catfish, Palu, Mali	OPPCP	Asthma, **spleen treatment**	Meat	Oral	75	13	0.17	17.33	1.00	17.33			0
26.	*Labeo gonius* (Hamiliton, 1822), Angra lebeo, Kala sarru	LGALKS	Joint pain	Meat	Oral	53	4	0.08	7.55	0.80	6.06			0

BPU (body parts uses), MOA (mode of application), FC (frequency of citation), MD (medicinal use), UV (use value), RPL (relative popularity level), FL (fidelity level), ROP (rank-order priority)

^*^Bold: Medical uses which are different than reported uses

### Use value (UV)

UV authenticates the relative importance of species. A comparative analysis of UV is given in Table 1. The highest UV of 0.86 was calculated for spotted snakehead (*Channa punctata*), whereas the lowest UV of 0.05 was found for karail fish (*Securicula gora*). The high UVs of fish species certified their constant use in the healing of various diseases.

### Relative popularity level (RPL)

**Fig. 5:**
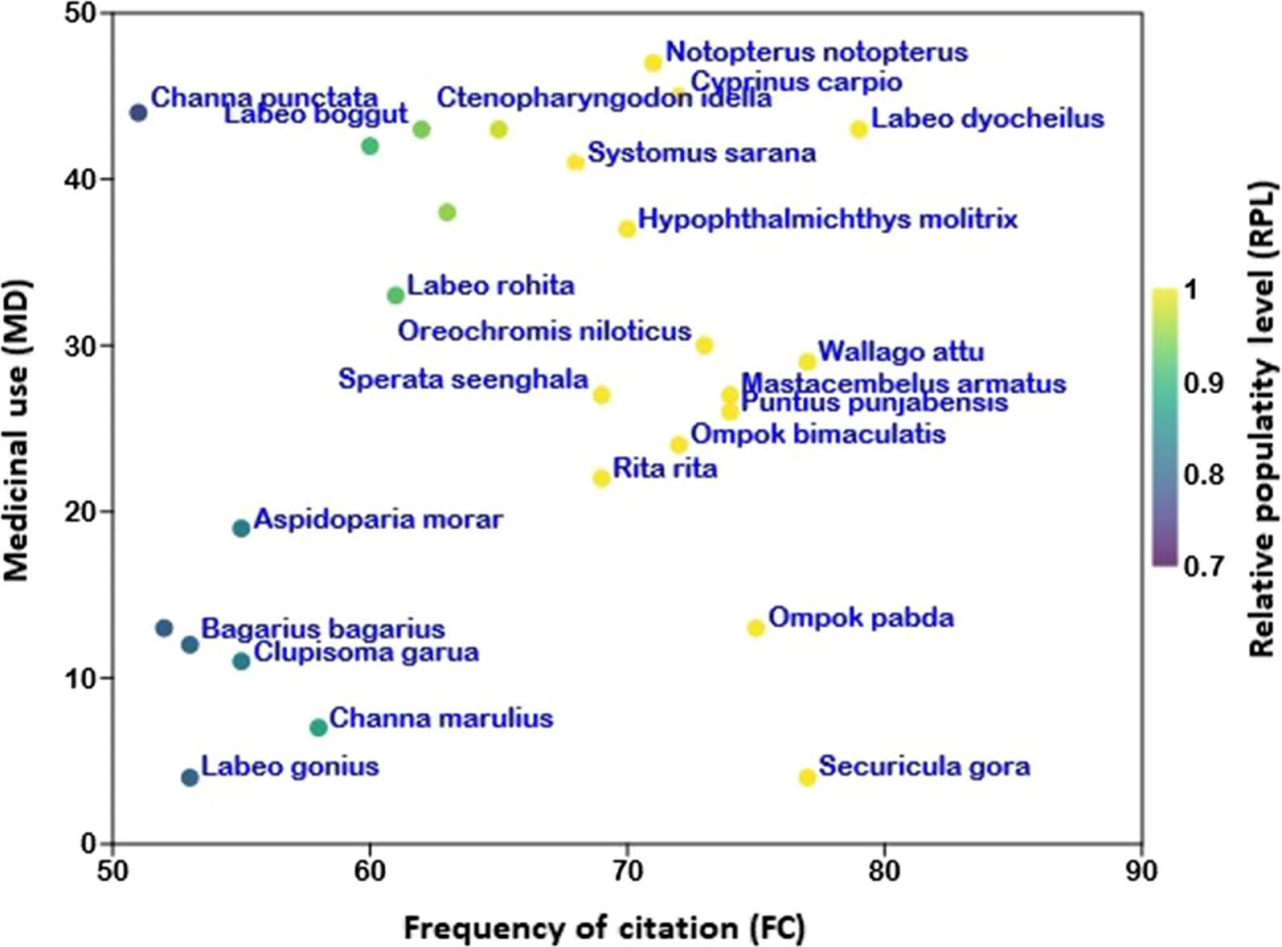
Relation between medicinal uses (MD), frequency of citation (FC), and relative popularity level (RPL) of fish species in southern Punjab, Pakistan. The circle color represents the RPL values. Based on RPL, fishes were divided into popular (RPL = 1.0) and unpopular (RPL < 1.0) groups

The relative popularity level (RPL) of the documented species is presented in Table 1 and ranged from 0.77 to 1.00. The species of fishes were divided into “popular” and “unpopular” groups based on RPL. These groups resulted were similar to Ali-Shtayeh, Yaniv [36] and Friedman, Yaniv [37]. During the survey, *Cyprinus carpio*, *Labeo rohita*, *Channa punctata*, *Oreochromis niloticus*, *Wallago attu*, *Hypophthalmichthys molitrix*, *Rita rita*, *Sperata seenghala*, *Notopterus notopterus*, *Labeo dyocheilus*, *Systomus sarana*, *Puntius punjabensis*, *Securicula gora*, *Ompok bimaculatus*, and *Ompok pabda* were found the most popular species with RPL = 1.0, while remaining fauna was categorized as unpopular (Fig. 5).

### Fidelity level (FL)

**Fig. 6:**
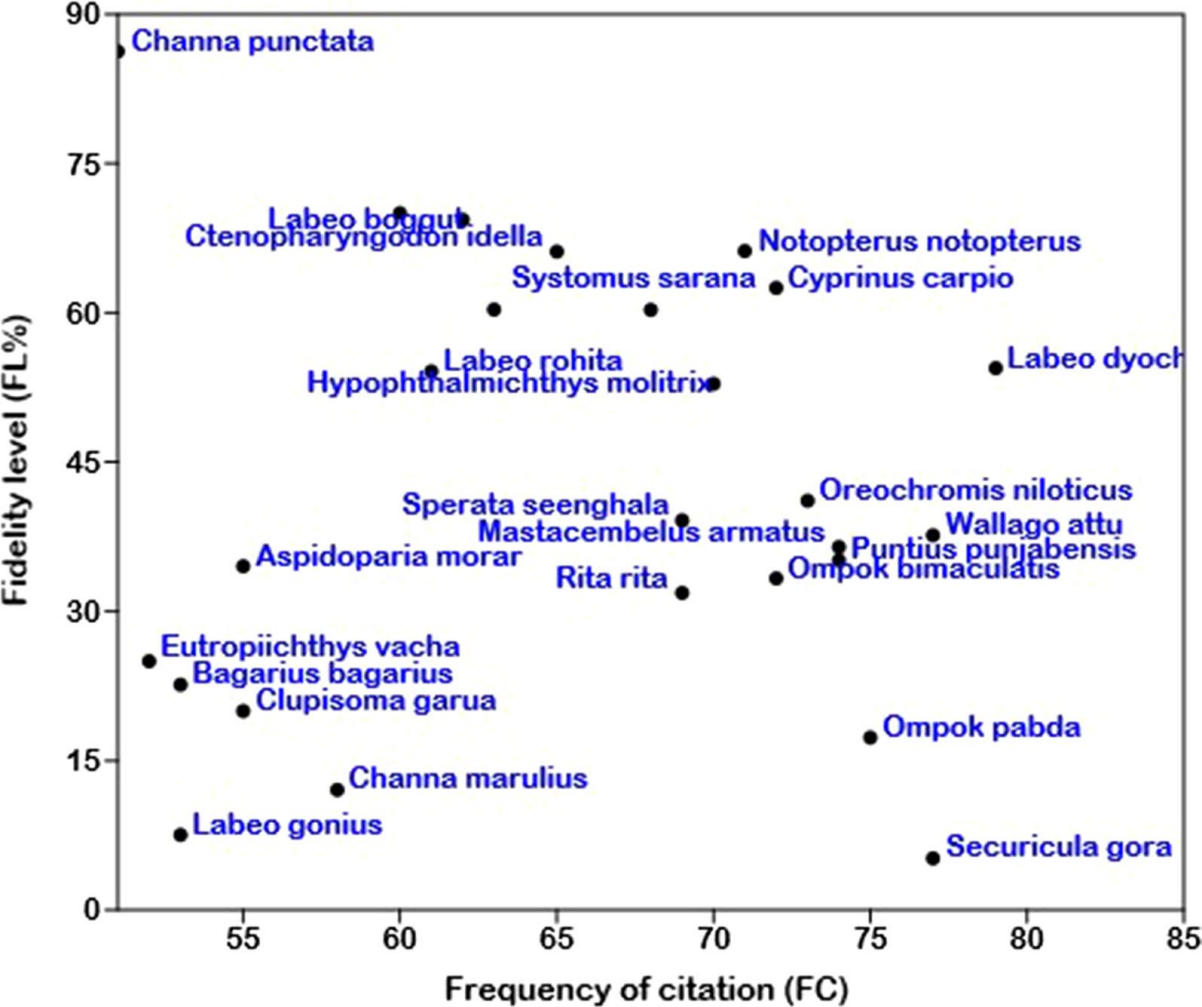
Relation between fidelity level (FL%) and frequency of citation (FC) of fish species in southern Punjab, Pakistan

The fidelity level is used to distinguish species of fishes that are generally preferred by local people to cure different illnesses [42, 43]. The FL of species of fishes in this research ranged from 5.19 to 86.27% (Table 1). Five species (*Ctenopharyngodon idella*, *Hypophthalmichthys molitrix*, *Cyprinus carpio*, *Channa punctata*, and *Oreochromis niloticus*), which were applied for backbone pain, cold, cough, eyesight, impotency, joint pain, night blindness, skin burn, and weakness, had more than 60% FL (Fig. 6).

### Rank-order priority (ROP)

**Fig. 7:**
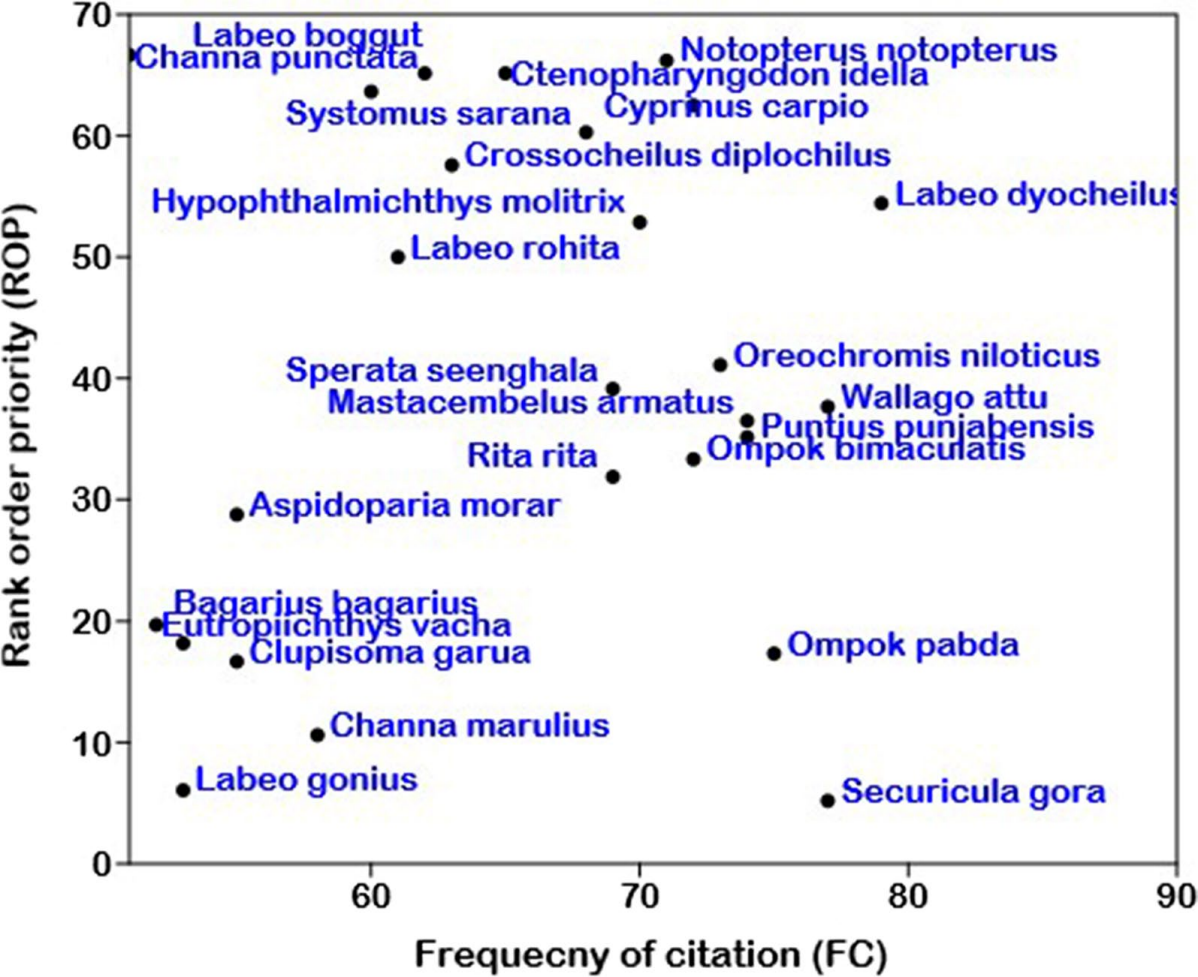
Relation between rank-order priority (ROP) and frequency of citation (FC) of fish species in southern Punjab, Pakistan

The categories of ROP are shown in Fig. 7. The ROP of five species was above 60. *Ctenopharyngodon idella*, *Cyprinus carpio*, *Notopterus notopterus*, *Labeo boggut*, *Systomus saran*, *Mastacembelus armatus*, *Channa punctata*, and *Oreochromis niloticus* were utilized to treat backbone pain, cold, joint, cough, eyesight, impotency, joint pain, night blindness, skin burn, and weakness.

## Discussion

### Socio-demographic data

Gathering socio-demographic data is critical in ethno-ichthyological research because it plays a vital role in analyzing and characterizing factors related to therapeutic and cultural applications of fish species. Educated people of the study region were less familiar with the usage of different fish species to cure common ailments because of their greater exposure to modernity. During the fieldwork, it was recorded that un-educated people (*N* = 29) have more ethnomedicinal knowledge as compared to educated people. Un-educated people use fish products commonly. Because they prefer to treat themselves rather than seek advice from local health practitioners or doctors. Similar findings were found in Ethiopia [44, 45] and Thailand [46]. We noted that rural people had less knowledge about the conservation and sustainable use of species as compared to urban ones. Gathering socio-demographic data on respondents (gender, age, educational level, occupation, and ethnicity) is particularly beneficial in social research as this element plays a significant role in analyzing and interpreting the responses received [47].

### Local nomenclature

Local names of fauna are generally depending on associations, ecology, habitat, morphology, and relation of species with humans. Vernacular names of six species included the same suffix “carp” (*Ctenopharyngodon idella* (grass carp), *Hypophthalmichthys molitrix* (silver carp), *Labeo boggut* (minor carp), *Cirrhinus reba* (reba carp), *Cirrhinus mrigala* (mrigal carp), *Cyprinus carpio* (common carp)). Similarly, five species had the suffix “machhali,” viz. *Oreochromis niloticus* (tilapia/chira machhli), *Mastacembelus armatus* (baam machhali), *Labeo calbasu* and *Aspidoparia morar* (dahi machhli), and *Securicula gora* (karail machhali). The English as well as vernacular names of *Hypophthalmichthys molitrix* was the same ("silver carp”), *C. idella* “grass carp,” *Labeo boggut* “minor carp,” *Cirrhinus reba* “reba carp” *Cirrhinus mrigala* “mrigal carp,” *Cyprinus carpio* “common carp.” The name of the four species reflected their color: *C. idella* has grass color, thus was called “grass carp” and *H. molitrix* has silver color and thus called “silver carp” *Gonialosa manmina* has golden color and called “golden sarru,” while *Labeo gonius* has black color and was called “kala sarru.” On the other hand, some fishes were locally classified based on structure, viz. *Oreochromis niloticus* (tilapia/chira machhli) appears like a house sparrow and was called “chira machhli” (chira meaning house sparrow), and the shape of “*Channa punctata”* is identical to an arm muscle so this species was known as “dola” (mean is muscle), “*Channa striata”* and “*Channa marulius”* appear just like the “sole of shoes” and were called “sole” (Additional file 1: Table S1).

### Cultural values of fish

**Fig. 8:**
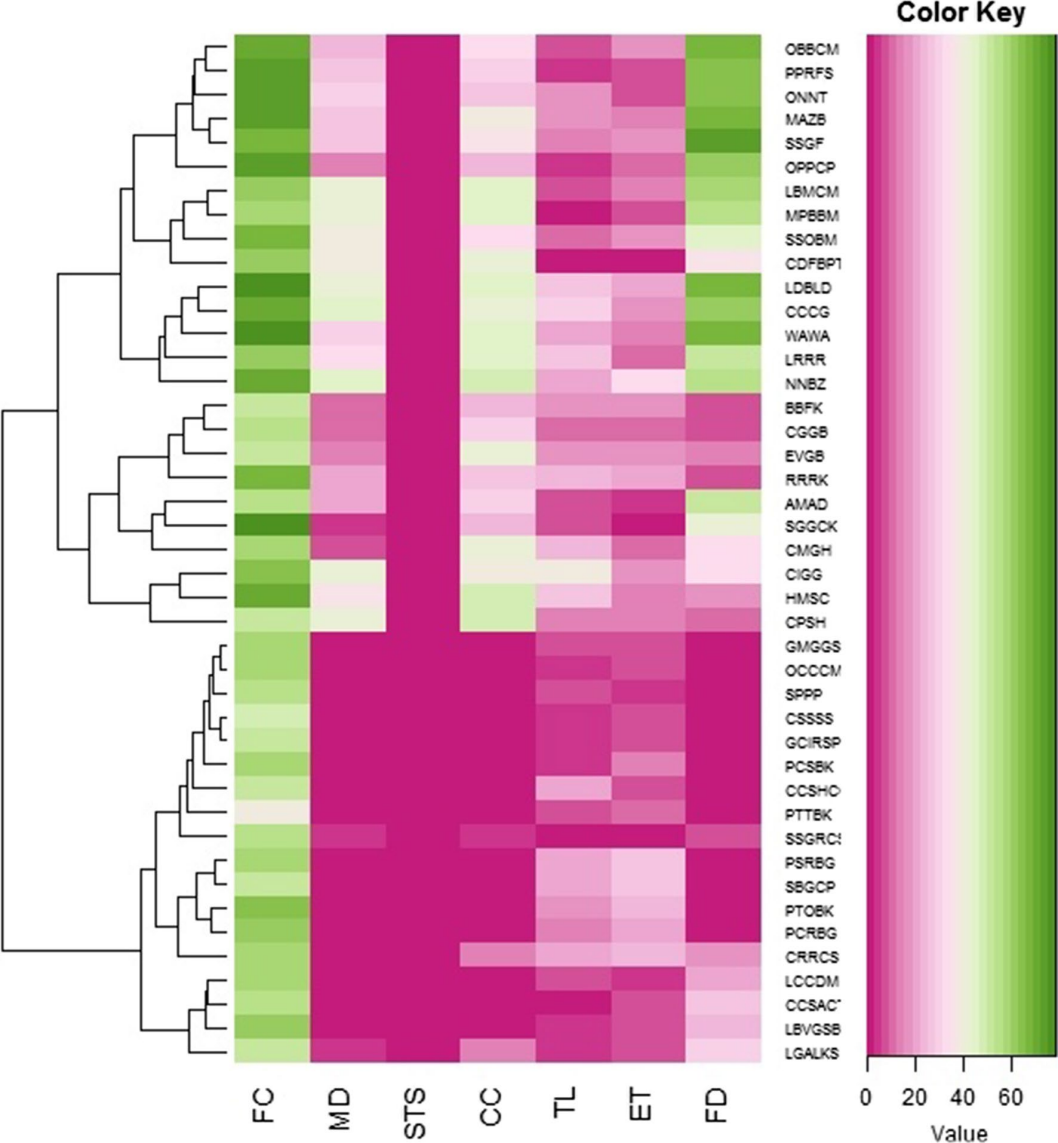
Cultural uses of fishes of the study area, FC (frequency of citation), MD (medicinal), STS (superstitious), CC (commercial) TL (tool), ET (entertainment), and FD (food), codes are present in Additional file 1: Table S1. Green and purple colors indicate increased and decreased values of informants, respectively

Fish were not only utilized in the therapy of diseases but also other purposes, e.g., fish for food or source of recreation, fish oil as a dietary supplement, fish as a biological control agent, or reference to fish in folklore, mythology, religion, spirituality, art, literature, film, and popular culture [10, 17, 48–50]. The local people of the study area also utilized meat of fish species collected from the Indus River of southern Punjab, Pakistan (Fig. 8). Medicinal and cultural purposes can be addressed by ecological factors such as resource availability, species status in the food chain, or the relevance of these species in the economy and social relationships within the community.

#### Fish as foods

Out of 43 fishes fauna, only *Gudusia chapra* (palla), *Gonialosa manmina* (golden sarru), *Puntius sophore* (gulfam), *Puntius conchonius* (gulfam), *Puntius ticto* (khirni), *Puntius terio* (khirni), *Puntius chola* (khirni), *Salmophasia bacaila* (popal), *Salmophasia punjabensis* (popal), *Osteobrama cotio* (chan makhni), *Chela cachius* (chan makhni), and *Channa striata* (soul) were not utilized in food of local people, while all other fishes were consumed as a food (Fig. 8). Muslims make up the majority of South Punjab's population, which eats fish often. Because fish and seafood are notably significant in Islamic traditions, especially when halal is used as a dietary criterion. Fish and other aquatic creatures do not have to be slaughtered religiously, albeit the technique employed must be humane, and no blessing must be offered. A fish that dies naturally and is floating or laying on the surface of the sea is still halal if it shows no evidence of decomposition or decay. People consume fish in a variety of ways such as smoked fish, barbeque fish, curry fish, and fried fish. For example, the inhabitants of Kashmir liked to eat barbequed fish in the evening hours [50]. They also used different traditional preservation techniques for fish food conservation e.g., smoking, pickling, and sun drying.

*Wallago attu* is a large freshwater catfish found in Pakistan that is popular as a food source in southern Punjab. The quick growth and good nutritional content of this great food fish prompted research into its aquaculture potential [51]. Catfish, for example, is a popular food in the fishing community, indicating a complex interaction of symbolic and cultural aspects, as well as materialistic or practical factors, such as the region's availability of this resource [52]. Hasan, Ahmad [53] reported that some fishes from the river Swat, Pakistan, such as *Racoma labiata*, *Schizothorax plagiostomus*, *Mastacembelus armatus*, *Tor macrolepis*, *Cyprinus carpio* and trout species are consumed as food hence deemed more economically important.

Fisheries generally play an important role in the global provision of food [54], directly accounting for at least 15% of the animal protein consumed by humans and indirectly supporting food production through aquaculture and livestock industries [55]. Meat and fish have been the primary source of protein for many human cultures throughout history [56]. There is significant potential for fisheries development in Pakistan, and substantial fish resources may be utilized as an essential source of high-quality protein meals. Increased fish production and consumption would boost the protein composition of people's diets [57]. According to the Tacon and Metian [58], over 75% of global fish production in 2002 is used directly for human consumption, and consumption of fresh fish is growing at the expense of other forms of fish products (e.g., canned fish).

#### Superstitious stories about fish

According to the local people, if one has body parts of the giant river catfish (*Sperata sarwari*) at home, black magic will not affect any person. In Latin American literature, we find numerous examples of animals giving “signs” of future happenings of a social nature, or animals known as being “of ill omen” [59] as is the case of black cats [60]. In his book on Mapuche secrets and legends, Calvo [61] wrote that eating fish would bring bad luck. Contrastingly, watching fish in an aquarium can reduce stress [62] and anxiety [63], improve physical mobility, reduce blood pressure in patients with cardiac disease [64], and confer physical benefits such as weight gain to older persons with dementia [65]. According to Alves and Rosa [66], the inhabitants of Brazil used tarpon fish scales to repulse evil eye, eliminate harassing spirits, block harmful influence, aphrodisiac, and treat asthma. In another research, Djidohokpin, Sossoukpè [52] African cryptic snakehead fish is frequently employed as a magical weapon by traditional healers to keep women from committing adultery. According to Neuenschwander and Sinsin [67], the magical characteristics of some sorts of fish can operate as an aphrodisiac.

#### Commercial use

Twenty-seven species of fishes were utilized for commercial purposes, i.e., *Aspidoparia morar*, *Bagarius bagarius*, *Channa marulius*, *C. punctata*, *Clupisoma garua*, *Crossocheilus diplochilus, Ctenopharyngodon idella*, *Cyprinus carpio*, *Eutropiichthys vacha*, *Hypophthalmichthys molitrix*, *Labeo boggut*, *L. calbasu, L. dyocheilus*, *L. gonius*, *L. rohita*, *Macrognathus pancalus*, *Mastacembelus armatus*, *Notopterus notopterus*, *Ompok bimaculatus*, *O. pabda, Oreochromis niloticus*, *Puntius punjabensis*, *Rita rita, Securicula gora*, *Sperata seenghala*, *Systomus sarana,* and *Wallago attu* (Fig. 8). For commercial purposes, the local people of the study area captured fish and trade to fulfill socioeconomic needs. Many fish species are traded and sold as curiosities (curios) and souvenirs around the world, either dried or preserved, whole or in part [68]. The inhabitants of the area used fish skin to create several consumer products including wallets, belts, and gloves. Taxidermy fish were also used as attractions in a variety of businesses. In Chinese tradition, ground fish bones are used to make toothpaste [69]. According to Blades [70], several types of Chinese drums (*ku*) are made from long pieces of bamboo or wood with dried fish skin stretched over one end.

#### Tools

According to local people of southern Punjab, all fingerlings of fish species (*N* = 43, Fig. 8) were also utilized as a tool to capture other fish when their sizes are very small [71]. Local inhabitants used fish flesh as bait for varieties of fish species from rivers, as also reported earlier by Altaf, Abbasi [10]. Additionally, bait fish are tiny fish that fishermen catch and use as baits to catch larger predatory fish, particularly game fish [52]. Typically, baitfish species are ones that are widespread and reproduce quickly, making them simple to capture and in plentiful supply. Fish parts have also been used in many ways to construct tools and weapons in different cultures. For example, dried Pirarucu tongues are traditionally used as a grater for mandioca root to produce cassava flour in many Amazon villages [69]. In Australian aboriginal culture, Kaiya was composed of an *Acacia* shaft mounted with a cluster of fish tail spines and used in initiation ceremonies, for fighting and punishment, such as spearing the leg of those who disobeyed tribal laws [72].

#### Entertainment

All 43 species found were utilized for entertainment and enjoyment (Additional file 1: Table S1). Most species are employed in therapeutic medicine to heal ailments or for mysterious rituals; therefore, catching them is great entertainment and enjoyment for the fisherman. Local people liked fishing in the area for recreation and enjoyment. Recreational fishing contests are a relatively new idea in which fishermen compete for rewards based on the total weightage of fish caught in a specific period of time. Competitive sport fishing has grown in popularity over the past several decades [73, 74]. Hasan, Ahmad [53] observed that several of the tiny-sized fishes from the Swat River in Pakistan, such as *Puntius*, *Barilius*, *Schistura*, and *Colisa*, are attractive ornamental species that are kept alive in aquaria and have a high economic value. If properly marketed, these fish may minimize the national spending on ornamental fish imports.

### Ethnomedicinal uses of fishes

**Fig. 9:**
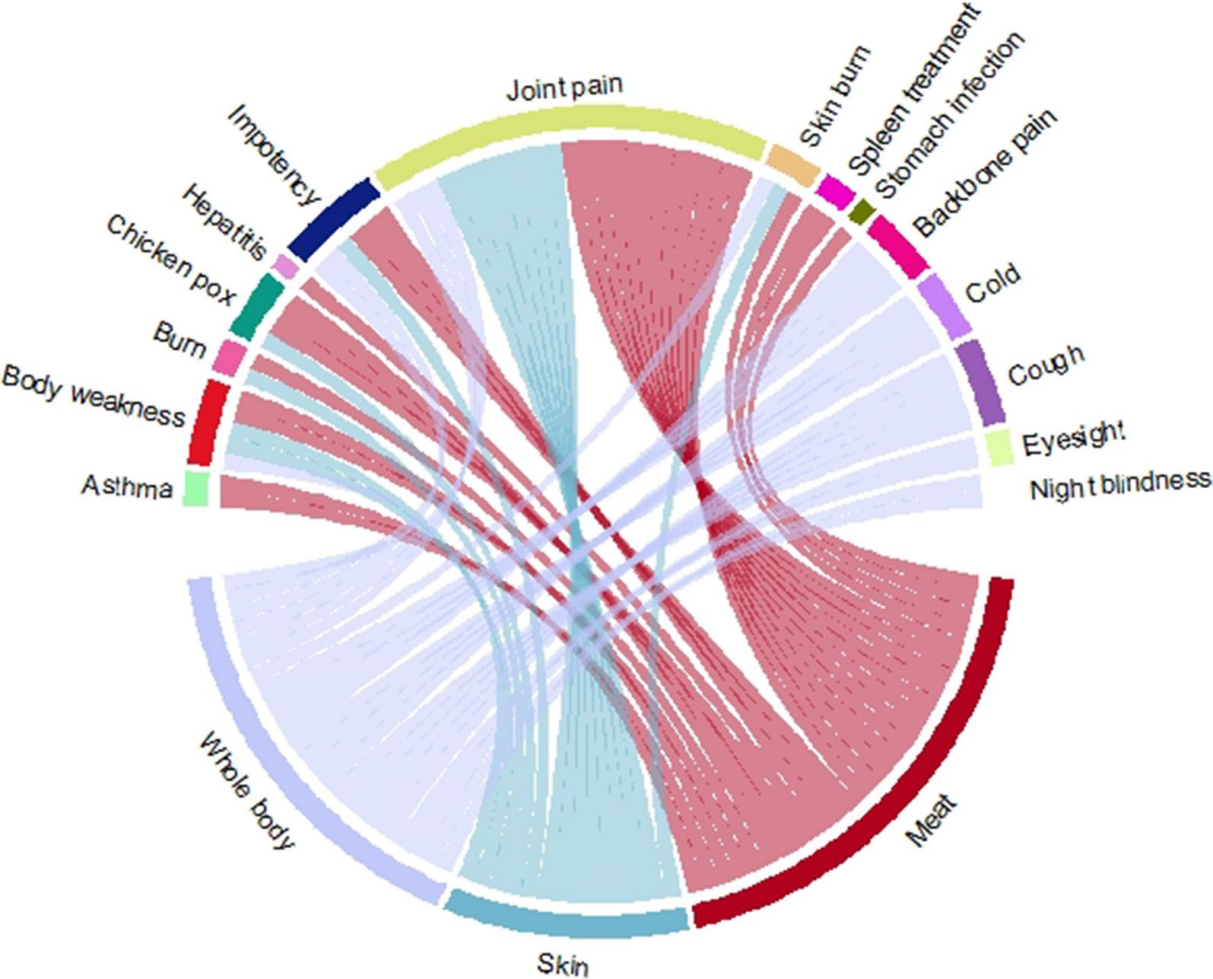
Body parts’ uses against diseases in the study area

South Punjab residents had detailed information about cultural as well as folklore medicinal uses of fish species. A total of 26 fish species were utilized to treat a wide variety of illnesses such as asthma, body weakness, burn, chicken pox, cold, cough, eyesight, hepatitis, impotency, joint pain, night blindness, skin burn, spleen treatment, stomach infection, and weakness (Table 1 and Fig. 9).

#### Body part(s) utilized

The meat was the most regularly consumed part and was utilized in 19 treatment recipes, followed by oil, brain, and skin, utilized in 10, 7, 13 recipes, respectively (Fig. 9).

During the present study, fish meat was utilized to cure eyesight, cough, cold, joint pain, backbone pain, night blindness, impotency, weakness, skin burn, chicken pox, and hepatitis. The oil of fish was utilized to cure body weakness, chicken pox, skin burn, joint pain, and impotency. Whole fishes were utilized to cure eyesight, cough, cold, night blindness, joint pain, backbone pain, impotency, body weakness and skin burn, while the skin only served to cure body weakness, chicken pox, skin burn, joint pain, and impotency (Table 1).

#### Diseases treated

**Fig. 10:**
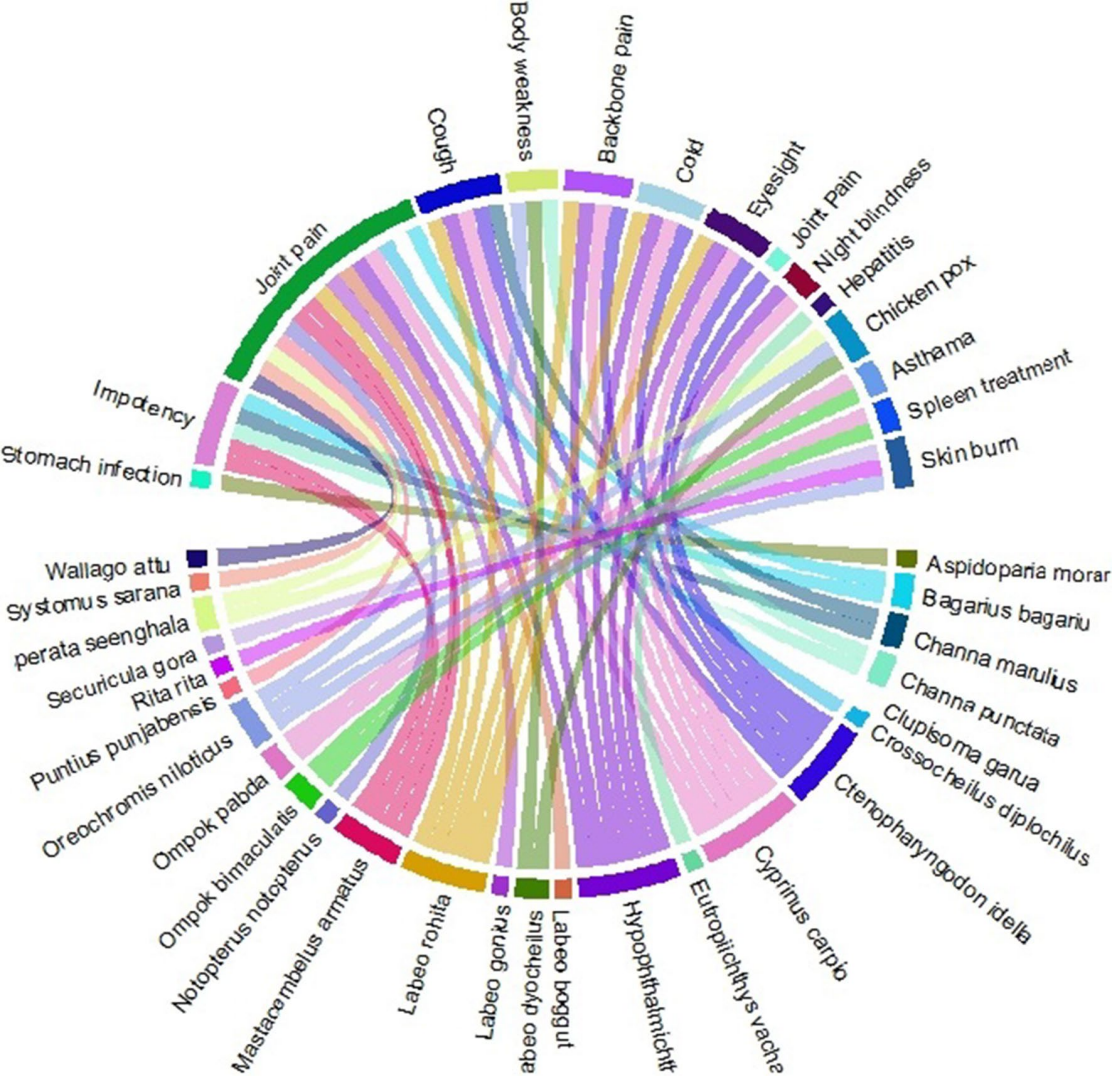
Fish species’ distribution according to the usage of different parts in southern Punjab Province, Pakistan

People used *Ctenopharyngodon idella* for the healing of cough, cold, joint, eyes problems, and backbone pain (Fig. 10), while previous studies documented this species for treating the central nervous system (CNS) disease, joint pain, and impotency [75, 76]. In another research, *Cyprinus carpio* was utilized for the healing of eyesight, cough, cold, joint and backbone problems, and the same species has been known to treat lumbago, memory, central nervous system disease, erysipelas, sexual problems, energy, overweight problems as well as cold [49, 76]. Likewise, *Labeo rohita* was utilized for the healing of eyesight, cough, cold, joint and backbone pain, and previously recorded to treat weakness, rheumatic problems, urine problems, stomachache, memory, energy, cold and sexual problems [76–79].

*Channa marulius* was used for cough and impotency, while it has been reported to treat memory, sexual issues, energy, rheumatic, cold, and hemoglobin [20, 77, 78, 80]. *Channa punctata* was utilized for the healing of impotency and weakness and the same species was documented previously to treat enhanced energy, pain, sexual issues, and joint problems [76, 78, 81, 82]. *Oreochromis niloticus* is used for healing body weakness, chicken pox, and skin burns and was used in other regions for eyesight, scorpion bites, abscesses, carbuncle, memory, sexual problems, and energy (44, 65).

*Rita rita* was used for the healing of skin burns, and has been known to cure joint problems, sexual problems, treat cold, joint pain and energy [76, 77]. *Bagarius bagarius* was utilized to cure joint issues and impotency (Table 1), while the same species was previously reported to treat body burns, body pain, stomach pain [78, 83]. *Wallago attu,* in the study region preferred for the healing of joint problems, was also mentioned in the literature for the same purpose, as well as piles, memory disorders, dysentery, liver and sexual problems, and cold [76, 84–86]. *Notopterus notopterus* was utilized for the healing of joint problems, in contrast to earlier reported uses to cure pain and chicken pox [87, 88].

The most popular species with RPL = 1.0, i.e., *Hypophthalmichthys molitrix*, *Mastacembelus armatus*, *Sperata seenghala*, *Eutropiichthys vacha*, *Clupisoma garua*, *Labeo dyocheilus*, *Labeo boggut*, *Systomus sarana*, *Puntius punjabensis*, *Crossocheilus diplochilus*, *Labeo gonius*, *Aspidoparia morar*, *Mastacembelus armatus*, *Ompok bimaculatus*, and *Ompok pabda*, were utilized to treat asthma, backbone pain, blindness, chicken pox, cold, cough, eyesight, hepatitis, impotency, joint pain, night, spleen healing, and stomach infection.

### Similarity index

Twenty-six species overall were utilized in traditional medicine (Table 1). Out of this, only one species (*Clupisoma garua*, garua bachcha) reached a similarity index of one. Its meat, skin, and oil were utilized to treat joint pain. Similar discriptions were reported by Altaf, Abbasi [10]. *Hypophthalmichthys molitrix* (silver carp) had a similarity index of 0.83 and was utilized to treat cold, night blindness, eye problems, cough, joint and backbone pain, while previous studies reported its use for fever, eyesight, cough, cold, and backbone pain [10, 17]. *Ctenopharyngodon idella* (grass carp) had 0.25 similarity and was used to treat eyesight, cough, cold, joint and backbone pain. Earlier studies had documented its use for sexual power, joint pain, backbone pain, enhance memory, energy and cold [10, 75, 76]. *Wallago attu* (wallago catfish) showed a similarity of 0.12 and was used to treat joint pain., very different from previous research that mentioned its use as liver tonic, for piles, dysentery, memory, liver, cold, sexual problems, and joint problems [76, 84–86]. *Cyprinus carpio* (common carp) had a 0.08 similarity index and served for eye problems, cough, cold, night blindness, joint issues, backbone pain, before the species was already known for increasing sexual power, treat overweight, lumbago, erysipelas, memory, energy, cold, and central nervous system disease [49, 76]. *Labeo rohita* (rohu) had a similarity of 0.07 and was used to treat eyesight, cough, cold, joint and backbone pain, while previously it was reported as remedy for weakness, rheumatic pain, cold, urine problem, and stomachache, enhancing memory, energy, and sexual power [17, 76–79].

Twenty species had a “zero” similarity index, and of the medicinally used species eight (i.e., *Channa marulius*, *Channa punctata*, *Oreochromis niloticus*, *Rita rita*, *Bagarius bagarius*, *Mastacembelus armatus*, *Eutropiichthys vacha* and *Notopterus notopterus*) had “0” similarity. These species were known for their use for central nervous system disease, abscesses, appetite, blood purification, body pain, carbuncle, chicken pox, cold, energy, enhance memory, hemoglobin, joint pain, malaria, body pain, rheumatic pain, scorpion bite, sex power, stomach pain, vision, and weakness [10, 17, 19, 20, 49, 76–78, 80–83, 87, 88]. The medicinal use of *Labeo dyocheilus*, *Labeo boggut*, *Systomus sarana*, *Puntius punjabensis*, *Aspidoparia morar*, *Securicula gora*, *Crossocheilus diplochilus*, *Mastacembelus armatus*, *Ompok bimaculatus*, *Ompok pabda*, *Labeo gonius*, and *Sperata seenghala* was recorded for the first time for body weakness, stomach infection, skin burns, joint pain, impotency, asthma, spleen healing and chicken pox.

## Conclusion

Traditional ethnomedicinal uses of 26 fishes were documented, and to best of our information the traditional medicinal uses of 20 species had “zero” similarity index, and 12 species included *Labeo dyocheilus* (body weakness), *Labeo boggut* (joint pain), *Systomus sarana*, *Puntius punjabensis* (joint pain), *Aspidoparia morar* (stomach infection), *Securicula gora* (skin burn), *Crossocheilus diplochilus* (joint pain), *Mastacembelus armatus* (impotency), *Ompok bimaculatus* (asthma), *Ompok pabda* spleen treatment, *Labeo gonius* (joint pain), and *Sperata seenghala* (joint pain) which were recorded first time. Fish are not only utilized in the therapy of diseases but also for other purposes, e.g., fish for food or amusement, fish as a tool, or connections to fish in folklore, mythology, religion, and spirituality. For instance, freshwater catfish was discovered to be a most popular food among fishermen, showing a complex combination of symbolic and cultural components. Our findings showed that the local people of the study area hold noteworthy traditional knowledge about the medicinal and cultural uses of fish species. Further study should concentrate on the differences in the ethno-ichthyological knowledge of this area to protect and conserve their records, which might be useful for the sustainable use, management, and conservation of the local ichthyofauna in southern Punjab, Pakistan. Furthermore, comprehensive analysis of active chemicals and in vivo and/or in vitro from fishes with elevated FC and UVs could be interesting for research on new drugs.

## Supplementary Information

**Additional file 1. Table S1:** Cultural information collected from local people and status of fishes.

## Data Availability

All the data are presented in tables and figures in the article or as a supplementary material, and further inquiries can be directed to the corresponding authors.
