# Supplementary material for: Cross-cultural diversity analysis: traditional knowledge and uses of freshwater fish species by indigenous peoples of southern Punjab, Pakistan
Source: J Ethnobiol Ethnomed. 2023 Jan 9;19:4. doi: 10.1186/s13002-022-00573-1 (PMC9827695; doi:10.1186/s13002-022-00573-1)
Supplement: Supplementary file 1 — Additional file 1. Table S1: Cultural information collected from local people and status of fishes. [file 13002_2022_573_MOESM1_ESM.docx]

**Table S1: Cultural information collected from local people and status of fishes**

| **Sr.** | **Scientific, common and local name** | **Status** | **Code** | FC | **MD** | **STS** | **CC** | **TL** | **ET** | **FD** |
| --- | --- | --- | --- | --- | --- | --- | --- | --- | --- | --- |
| 1 | *Ctenopharyngodon idella* (Valenciennes, 1844), Grasscarp, Grass carp | NE | CIGG | 65 | 43 | 0 | 41 | 38 | 16 | 32 |
| 2 | *Hypophthalmichthys molitrix* (Valenciennes, 1844), Silver carp, Silver carp | NT | HMSC | 70 | 37 | 0 | 49 | 26 | 13 | 18 |
| 3 | *Cyprinus carpio* (Linnaeus, 1758), Commoncarp, Gulfam | VU | CCCG | 72 | 45 | 0 | 44 | 31 | 16 | 61 |
| 4 | *Labeo rohita* (Hamilton, 1822), Rohu, Raho | LC | LRRR | 61 | 33 | 0 | 47 | 28 | 10 | 53 |
| 5 | *Channa marulius* (Hamilton, 1822), Great snakehead, Soul | LC | CMGH | 58 | 7 | 0 | 43 | 24 | 12 | 33 |
| 6 | *Channa punctata* (Bloch, 1793), Spotted snakehead, Dola | LC | CPSH | 51 | 44 | 0 | 48 | 14 | 13 | 11 |
| 7 | *Oreochromis niloticus* (Linnaeus, 1758), Nile tilapia, Chira machhli | LC | ONNT | 73 | 30 | 0 | 27 | 17 | 8 | 66 |
| 8 | *Rita rita* (Hamilton, 1822), Rita, Khaga | LC | RRRK | 69 | 22 | 0 | 28 | 24 | 19 | 9 |
| 9 | *Bagarius bagarius* (Hamilton, 1822), Goonch, Foji Khaga | NT | BBFK | 53 | 12 | 0 | 25 | 18 | 16 | 7 |
| 10 | *Mastacembelus armatus* (Lacepède, 1800), Zig-zag eel, Baam machhali | LC | MAZB | 74 | 27 | 0 | 39 | 16 | 15 | 68 |
| 11 | *Sperata seenghala* (Sykes, 1839), Giant river-catfish, Sangari | LC | SSGF | 69 | 27 | 2 | 36 | 14 | 16 | 73 |
| 12 | *Wallago attu* (Bloch & Schneider, 1801), wallago catfish, Mali | VU | WAWA | 77 | 29 | 0 | 47 | 22 | 13 | 69 |
| 13 | *Eutropiichthys vacha* (Hamilton, 1822), Batchwa vacha, Jhali | LC | EVGB | 52 | 13 | 0 | 44 | 16 | 17 | 13 |
| 14 | *Clupisoma garua* (Hamilton, 1822), Garua bachcha, Bachhwa | LC | CGGB | 55 | 11 | 0 | 31 | 12 | 10 | 7 |
| 15 | *Notopterus notopterus* (Pallas, 1769), Bronze featherback, But Pari | LC | NNBFBP | 71 | 47 | 0 | 49 | 19 | 33 | 54 |
| 16 | *Labeo dyocheilus* (McClelland, 1839), Brahmaputra Labeo, Dambra | LC | LDBLD | 79 | 43 | 0 | 47 | 28 | 22 | 69 |
| 17 | *Labeo boggut* (Sykes, 1839), Minor carp, Bogat | LC | LBMCM | 62 | 43 | 0 | 45 | 8 | 13 | 59 |
| 18 | *Systomus sarana* (Hamiliton, 1822), Olive barb, Sarana | LC | SSOBM | 68 | 41 | 0 | 34 | 12 | 17 | 47 |
| 19 | *Puntius punjabensis* (F. Day, 1871), Ray-finned fish, Silver fish | LC | PPRFS | 74 | 26 | 0 | 29 | 6 | 9 | 66 |
| 20 | *Aspidoparia morar* (Hamiliton, 1822), Aspidoparia, Dahi Machli | NE | AMAD | 55 | 19 | 0 | 29 | 7 | 4 | 51 |
| 21 | *Securicula gora* (Hamiliton, 1822), Gora-chela Gora chela, Karail fish | LC | SGGCK | 77 | 4 | 0 | 23 | 9 | 2 | 43 |
| 22 | *Crossocheilus diplochilus* (Heckel, 1838), Fringe barb, Pahari torki | NE | CDFBPT | 63 | 38 | 0 | 43 | 2 | 2 | 37 |
| 23 | *Macrognathus pancalus* (Hamiliton, 1822), Barred spiny eel, Baam Machhali | LC | MPBBM | 60 | 42 | 0 | 45 | 2 | 9 | 55 |
| 24 | *Ompok bimaculatus* (Bloch, 1793), Butter Catfish, Ompok machhli | NT | OBBCM | 72 | 24 | 0 | 33 | 9 | 16 | 69 |
| 25 | *Ompok pabda* (Hamiliton, 1822), Pabdah Catfish, Palu | NT | OPPCP | 75 | 13 | 0 | 25 | 6 | 12 | 63 |
| 26 | *Labeo gonius* (Hamiliton, 1822), Angra labeo, Kala sarru | LC | LGALKS | 53 | 4 | 0 | 13 | 6 | 9 | 29 |
| 27 | *Gudusia chapra* (Chaudhry,S. 2010), Indian river shad, Sarru, Palla | LC | GCIRSP | 51 | 0 | 0 | 0 | 4 | 7 | 0 |
| 28 | *Gonialosa manminus* (Hamiliton, 1822), Ganges gizzard, Golden sarru | NE | GMGGS | 57 | 0 | 0 | 0 | 7 | 7 | 0 |
| 29 | *Cirrhinus reba* (Hamiliton, 1822), Reba carp, Sarru | LC | CRRCS | 59 | 0 | 0 | 13 | 19 | 23 | 17 |
| 30 | *Catla catla* (Hamiliton, 1822), South Asian carp, Thaila | LC | CCSACT | 54 | 0 | 0 | 0 | 3 | 7 | 26 |
| 31 | *Labeo calbasu* (Hamiliton, 1822), Calbasu, Dahi Machli | LC | LCCDM | 57 | 0 | 0 | 0 | 9 | 4 | 21 |
| 32 | *Labeo bogga* (Hamiliton, 1822), Violet Gilled shark, Bhangri | LC | LBVGSB | 63 | 0 | 0 | 0 | 6 | 9 | 24 |
| 33 | *Puntius sophore* (Hamiliton, 1822), Pool barb, Barb machhali | LC | PSRBG | 57 | 0 | 0 | 0 | 21 | 27 | 0 |
| 34 | *Puntius conchonius* (Hamiliton, 1822), Rosy barb, Barb machhali | LC | PCRBG | 62 | 0 | 0 | 0 | 13 | 19 | 0 |
| 35 | *Puntius ticto* (Hamiliton, 1822), Ticto barb Two spot barb, Khirni | LC | PTTBK | 41 | 0 | 0 | 0 | 7 | 11 | 0 |
| 36 | *Puntius terio* (Hamiliton, 1822), Onespot barb or Teri barb, Khirni | LC | PTOBK | 65 | 0 | 0 | 0 | 18 | 23 | 0 |
| 37 | *Puntius chola* (Hamiliton, 1822), Swamp barb or chola barb, Khirni | LC | PCSBK | 59 | 0 | 0 | 0 | 5 | 13 | 0 |
| 38 | *Salmophasia bacaila* (Hamiliton, 1822), Gora chela, Popal | LC | SBGCP | 52 | 0 | 0 | 0 | 20 | 26 | 0 |
| 39 | *Salmophasia punjabensis*(F.Day, 1871), Punjab, Popal | NE | SPPP | 55 | 0 | 0 | 0 | 9 | 4 | 0 |
| 40 | *Osteobrama cotio* (Hamiliton, 1822), Cotio hafua, Chan makhni | LC | OCCCM | 57 | 0 | 0 | 0 | 4 | 7 | 0 |
| 41 | *Chela cachius* (Hamiliton, 1822), Silver hatchet chela, Chan makhni | LC | CCSHCC | 53 | 0 | 0 | 0 | 19 | 8 | 0 |
| 42 | *Channa striata* (Bloch, 1793), Striped snakehead, Soul | LC | CSSSS | 50 | 0 | 0 | 0 | 5 | 9 | 0 |
| 43 | *Chitala chitala* (F. Hamilton, 1822), Indian featherback, Chitala Machhali | NT | SSGRCS | 56 | 6 | 0 | 4 | 3 | 2 | 9 |

FC (Frequency of citation), MD (Medicinal use), (STS) Superstitious, CC (Commercial), TL (Tool), ET (Entertainment), FD (Food)**.**

*Status: NE (Not Evaluated), NT (Near Threatened), VU (Vulnerable), LC (Least Count)
